# Hallucinations and Illusions

**Published:** 1899-03

**Authors:** 


**Hallucinations and Illusions.** By EDMUND PARISH. Pp. xiv., 390. London: Walter Scott, Ltd. 1897.

This book, which is one of the "Contemporary Science Series," is an English edition of the German original, which grew out of an examination of the "International Census of Waking Hallucinations of the Sane," and forms, in the words of its sub-title, "A Study of the Fallacies of Perception" of great interest.

Psychology is still a very young science, and it is only within the last few years that it has taken its place as an accurate—and to a great extent experimental—study of facts. There is already an extensive literature on the subject, and Mr. Parish is evidently well acquainted with it. In fact, the work is the outcome of a great deal of research and careful reasoning. Dreams, hypnosis, fallacies of memory, association and dissociation, after-images, &c., are each dealt with in relation to hallucinations and illusions. From the evidence collected there can be no doubt of the comparative frequency of hallucinations amongst the healthy in a waking condition, and it is stated that it is "impossible in practice as in theory to distinguish between waking hallucinations and those of sleep." Physiology is naturally constantly referred to and modern theories of brain-function are clearly understood; but there are, here and there, allusions to the "subcortical centres" and "basal ganglia," which are perhaps not quite up to date in the *rôle* allotted to these parts.

A powerful case is made out against instances meant to prove telepathy or thought-transference, and this part of the book is of most interest to the general reader, because it is less technical and requires less previous knowledge of the subject.

There is a useful summary in the last chapter, and an appendix with carefully-prepared tables and illustrative cases at the end of the book.

The questions discussed in this "Study" are intricate and abstruse, and great praise must be given to the author for the pains he has taken to sift a large mass of evidence and bring his deductions clearly before the reader.

